# Expanding the early intervention offer: A new care pathway for children’s wellbeing practitioners in a south London child and adolescent mental health service

**DOI:** 10.1177/13591045231201195

**Published:** 2023-09-09

**Authors:** Lauren M Hickling, Julia Dabrowski, Sadie Williams

**Affiliations:** 1 Department of Psychology, King’s College London, 34426 Institute of Psychiatry Psychology & Neuroscience, London, UK; 2 4958 South London and Maudsley NHS Foundation Trust, Bethlem Royal Hospital, Beckenham, UK

**Keywords:** Child and adolescent mental health services, child mental health, mental health services, referral pathways, adolescent mental health, service development project

## Abstract

Child and Adolescent Mental Health Services (CAMHS) have been under recent increased demand, with increasingly limited resources, contributing to longer waiting lists, and a growing proportion of rejected referrals due to limited capacity and increasing thresholds. Child and Wellbeing Practitioners (CWPs) provide an opportunity to meet the needs of rejected referrals. We aimed to determine the feasibility of a new and direct referral route within a South London CAMHS. All referrals rejected to the local CAMHS in one year were assessed for inclusion to an embedded child and youth wellbeing in schools team (CYWS), and data collected on reasons for rejection, demographics and eligibility for the CYWS team. Of the 1,322 referrals made to CAMHS in this period, 317 were rejected. The most common reason for referral rejection was not meeting the severity threshold. One third of rejected referrals were judged to be eligible for inclusion to the CYWS team. Therefore, a significant number of children and young people (CYP) being rejected by CAMHS would be eligible for assessment and possible treatment under the CYWS team, making a new referral route potentially feasible, allowing more CYP to access mental health support and have a positive impact on waiting times.

## Introduction

It is estimated that 75% of mental health problems in adults emerge before the age of 18, with approximately 1-in-6 children and young people (CYP) aged 6–16 and 1-in-4 CYP aged 17–19 experiencing a probable mental health disorder (NHS Digital, 2021). Therefore intervening in the early stages is perceived as a priority (Murphy & Fonagy, 2012; Rocks et al., 2020; Rocks et al., 2020, 2020; Solmi et al., 2021; Solmi et al., 2021, 2021). Indeed, timely care is the basis for many healthcare models across the world (Edbrooke-Childs & Deighton, 2020). Early intervention is also an important focus for policy leaders and politicians, due in part, to the economic impact of poor mental health. Recent estimates suggest that untreated mental illness in adults costs around £105.2bn every year (Crena-Jennings & Hutchinson, 2020), and may impact subsequent costs to public services (Fonagy et al., 2014). Evidence has shown that young people who do not receive treatment have a significantly higher chance of needing help from adult services later in life (Fraser & Blishen, 2007) or of reaching crisis point (Care Quality Commission, 2017). Given the current long waiting times for children and adolescent mental health services (CAMHS) UK wide (Hansen et al., 2021), and the effects these can have on young people seeking support, consideration of the future service needs of CYP is an important step for future research and practice (Mind et al., n.d). This could partially be addressed through improvements to early intervention provision and the use of alternative treatment pathways.

### Child and adolescent mental health services: the current context

In January 2020, the Education Policy Institute (EPI) published a report into CAMHS access during the 2018/19 financial year. Twenty-six percent of referrals to CAMHS in England were rejected, which varied slightly by geographical area. During this period, 613,901 CYP were in contact with mental health services (NHS Digital, 2019) and the average waiting time was 50 days between referral and the start of treatment. The Five Year Forward report states that early intervention and prevention are cost-effective to implement (All-Party Parliamentary Group on Mental Health, 2018; NHS England, 2016). Investigations have found a gradual increase in the number of referrals and waiting times, perhaps in part due to the limited finances allocated to CAMHS from 2011 onwards. Additionally, the Five Year Forward report (NHS England, 2016) highlights that because of limited CAMHS capacity, high severity cases are prioritised. Therefore, vulnerable CYP in the early stages of illness may be unable to access support (All-Party Parliamentary Group on Mental Health, 2018), as they may not be seen as ‘severe enough’ to be accepted for support.

Even for the CYP who were accepted to CAMHS, reports have suggested that many are not getting the support they need while waiting to access their care (Care Quality Commission, 2017). Whilst accessing support via schools has been suggested as a possible complimentary form of help, a review of English schools showed that almost 50% of schools reported not offering counselling to pupils and 51% reported not having a designated mental health lead (Department of Health and Social Care & Department for Education, 2017). In recent years, there has been an attempt to fill the gaps in service provision for CYP and various initiatives have been implemented. For example, the Children and Young People Increasing Access to Psychological Therapies (CYP-MH, formerly known as CYP-IAPT) programme was developed in England in 2011 (Ludlow et al., 2020), and further support in schools was offered (Department of Health and Social Care & Department for Education, 2017, 2018). Initially, the programme aimed to train practitioners in outcome monitoring, evidence based psychological therapies and service-user involvement (Ludlow et al., 2020). The remit expanded over the next few years to expand the CAMHS workforce further, provide a low-intensity workforce and to pilot workforce trainees across a number of training sites and English schools, to bring child services broadly in line with adult IAPT services (Ludlow et al., 2020). Child and Wellbeing Practitioners (CWP) and Educational Mental Health Practitioners (EMHP) and were introduced to the CYP-MH workforce in 2017 and 2019 respectively, in order to provide school-based and community-based support respectively (Ludlow et al., 2020). However, while access to support from CAMHS practitioners based in schools can offer early intervention and a route into CAMHS for some CYP, this is highly dependent on geographical location, and sometimes based on which school a CYP attends.

## Aim & objectives

The aim was to determine whether there is scope to develop a new and direct referral route for CYP who do not meet current CAMHS thresholds, with the intention of allowing more CYP to receive mental health support from EMHPs and CWPs. The first objective in this original work was to identify how many CYP who are rejected for assessment by CAMHS would be suitable for treatment with a team of CWPs. Secondly, whether there were any significant differences in demographics between those who were rejected to a present CAMHS service. Finally, we aimed to explore the demographic characteristics of those who would be suitable for treatment with the CYWS team to determine whether any demographic groups would be disadvantaged by a potential change to the referral pathway. Neither of these aims have been empirically explored previously.

## Method

### Setting and participants

This pilot study was conducted within a CAMHS (part of South London and Maudsley NHS Foundation Trust) in a London borough which, in 2018 was home to over a third of a million people, with 21% of these being under the age of 20 years (Greater London Authority, 2021). It is an ethnically diverse area of London, with 41% of residents being from a BAME background and 37% being White British (Greater London Authority, 2021).

The CYWS team have three criteria for deciding who is eligible for intervention. Inclusion criteria include: (1) The CYP attends one of the schools linked to the CYWS team; (2) The referral fits with the team’s current treatment protocol, which assesses and provides interventions for mild to moderate anxiety and mild behavioural presentations in CYP of primary school age, as well as mild to moderate anxiety and depression presentations in CYP of secondary school or college age.

Exclusion criteria include: (1) currently seeking support from Children’s Social Care (excluding Early Help), or a presentation that suggests this support is indicated; (2) a CYP with a significant level of risk to self or others, including self-harm; (3) a high level of family discord/conflict or complicated environmental factors; and (4) evidence of the CYP having a significant learning disability without a co-existing mental health difficulty.

Referrals are made via a number of different sources. Thresholds for acceptance are high in tiers 2 and 3, in which the majority of accepted cases are moderate to severe in presentation (Department of Health and Social Care, 2015; NHS England, 2016). A duty worker (a staff member from the CAMHS team) reviews the referrals and completes a risk screen. Referrals are then reviewed at a Central Referrals Panel (CRP), before an outcome is given to accept or reject the referral. A mix of CAMHS professionals assess whether referred individuals meet the threshold for assessment and possible treatment along one of the care pathways provided by the service. This decision is based on several factors, such as the severity of the presenting difficulty, case complexity, and risk. Alternatively, whether signposting or re-referral to another service is warranted. If accepted to the service, CYP are then allocated to the waiting list prior to assessment, a waiting list prior to treatment, and finally allocation to a mental health practitioner. In the borough, just over 2,000 CYP were in contact with CAMHS between 2018 and 2019.

In November 2019, a new child and youth wellbeing in schools (CYWS) team was embedded within the current CAMHS to provide a mental health service to 5–18 year olds who do not meet the threshold for CAMHS acceptance. This team was embedded within eight local schools within the borough, from which they received their referrals. The team is comprised of CWPs, a senior clinical psychologist and a CAMHS specialist practitioner. More specifically, this team provide guided self-help to CYP and families using a Cognitive Behavioural Therapy (CBT) approach. The course of treatment usually consists of up to eight sessions of face-to-face, online or telephone work, lasting around 1 hour. The treatment usually consists of guided self-help which includes CBT-based psychoeducation, cognitive and behavioural strategies, and a staying well plan. Currently, CYP can be referred to the CYWS team via their teacher, Special Educational Needs Coordinator (SENCO), school support staff, or via self-referral from a parent or CYP (if ≥ 16 years old).

### Procedures

Data on all referrals to the present CAMHS via the CRP between September 2019 and September 2020 was analysed. For all young people referred to the service, the amount of time between referral and decision at CRP was calculated to give an indication of waiting time to a decision to accept or reject the referral to specialist CAMHS. Data available from the CRP database included age at the time of review at CRP, referral origin (coded as either General Practitioner (GP), Accident & Emergency (A&E), Social Services, Education, Community Health, Internal, Self-referral or Other). The primary reason for rejection to specialist CAMHS was collected by reviewing the referred individual’s electronic medical record, as well as ethnicity (where available). After combining data from electronic medical records and the CRP database, the authors determined whether each CYP rejected to the present CAMHS would have met the threshold criteria for inclusion to the CYWS team. If unsure, the lead researcher sought a second opinion from the team’s lead. Some CYP were referred multiple times during the time period analysed.

Data was analysed using SPSS v27 (IBM Corp). Data for all rejected referrals to specialist CAMHS was first analysed in its entirety, then analysed according to whether or not they were deemed as eligible for acceptance into the CYWS team. Categorical data is represented by frequencies and range, and continuous data by means and standard deviation (SD). For non-parametric data, a mode was calculated. Statistical significance was tested using independent samples t-tests for continuous data, and chi-square tests for categorical data.

## Results

Over the period from September 2019 to September 2020, 1,323 referrals were made to the CAMHS team. One of these referrals was excluded from further analysis as the associated electronic medical record could not be found, leaving 1,322 referrals (ranging from 36–187 referrals per month). A total of 317 referrals were rejected to the present specialist CAMHS during the same period (ranging from 4–46 rejections per month). Around 12.30% to 53.49% of referrals received monthly were rejected (Table 1).

**Table 1.** Demographic characteristics of rejected referrals to the present CAMHS between September 2019 and September 2020.

	Mode (Mean)
Age	13 & 16 years (11.52)
	Frequency (%)
Sex (Male)	176 (55.50%)

### Characteristics of rejected referrals

The ages of the CYP not meeting threshold for assessment in present CAMHS during this period ranged between 3–18 years old. The data was bi-modal at 13 (*n* = 28) and 16 (*n* = 28) years of age, with both falling within transition periods, at the beginning and end of secondary school respectively.

**Figure 1.**
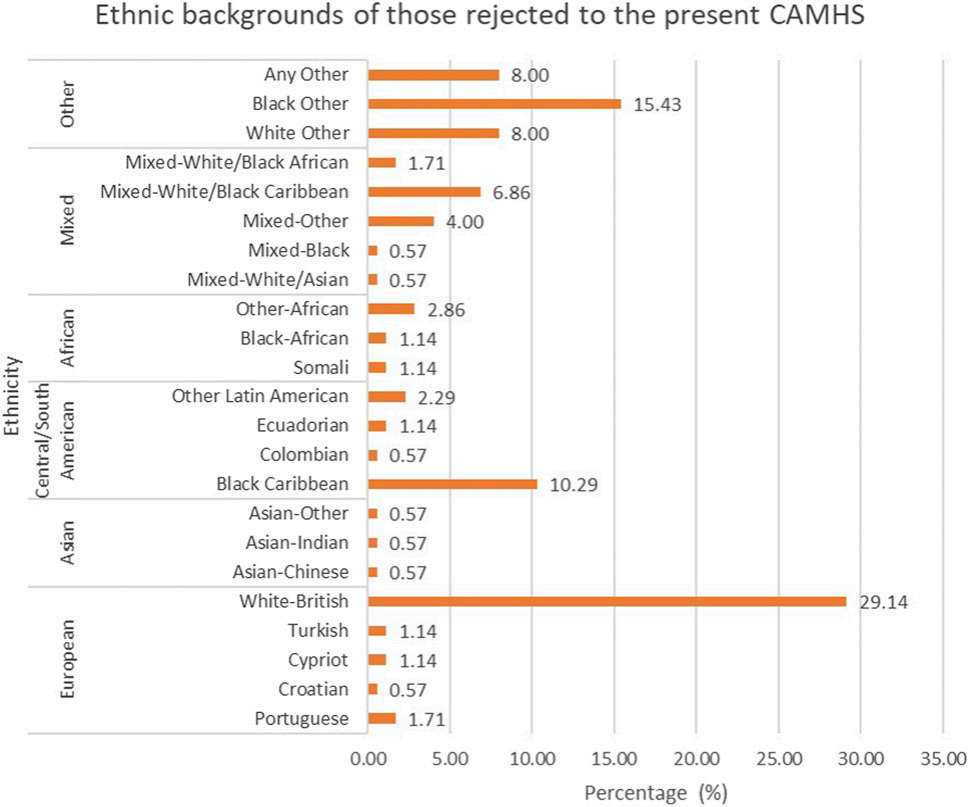
The ethnic distribution of referrals rejected to the present CAMHS between September 2019 and September 2020 (*N* = 175).

The ethnic distribution of these rejected referrals was wide, representing 23 ethnic groups, which can be observed in Figure 1. These figures differ from those officially observed in the wider borough (Greater London Authority, 2021), however this may in part be explained by the different categories used to define ethnic group. However, some groups had comparable proportions, for example, 8% of borough residents identify as Black Caribbean versus 10% who were rejected to the present CAMHS (Greater London Authority, 2021). Ethnicity was not recorded in a large proportion of CYP (44.8%). Of the referrals with a recorded ethnicity, 33.71% of these are from a European background, 1.71% from an Asian Background, 14.29% from a Central/South American background, 5.14% African, 13.71% from a Mixed ethnic background and 31.43% from another unspecified background (Figure 1).

### Referral route

As seen in Table 2, referrals to the present CAMHS were obtained via a number of different routes. Most referrals came from GP services (55.20%), with the majority of these being referred between October 2019 and March 2020, after which the number of referrals dropped to more consistent levels. Whereas referrals from educational institutions peaked in March 2020, when lockdown started in the UK, and continued consistently throughout the summer. This was despite school being remote for the majority of CYP, except from the children of key workers and those deemed clinically or socially vulnerable.

**Table 2.** The referral origin of rejected referrals to the present CAMHS, from September 2019 to September 2020.

	Frequency (%) *N* = 317
Referral route	
A&E	14 (4.40%)
Community health	45 (14.20%)
Education	52 (16.40%)
GP	175 (55.20%)
Internal	2 (.60%)
Self-referral	4 (1.30%)
Social services	21 (6.60%)
Other	4 (1.30%)

### Reasons for referral rejection to specialist child and adolescent mental health services

**Figure 2.**
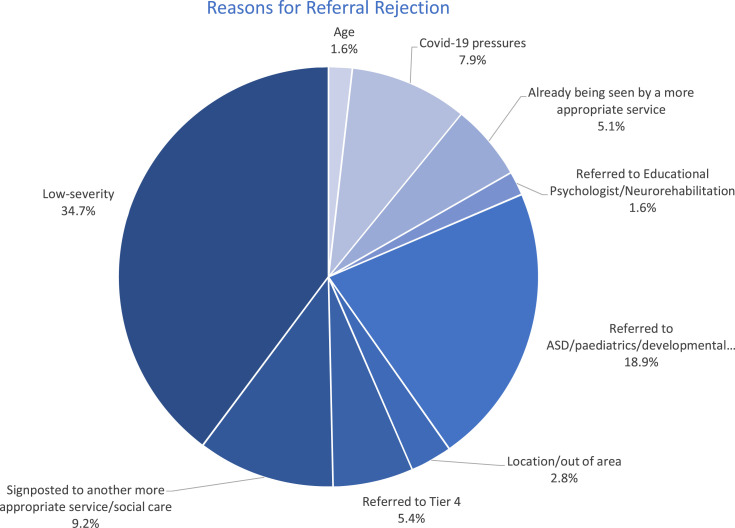
Reasons for referral rejection in CAMHS, from September 2019 to September 2020.

The most common reason for a referral being rejected was that the CYP’s presentation did not meet the threshold for symptom severity for inclusion into a Tier II/III service (targeted CAMHS services or specialist CAMHS respectively (Department of Children Schools & Families, 2008)). See Figure 2 for reasons for referral rejection. Being deemed to be too low in severity was the case for 110 referrals received in the studied time period (34.70%). The second most common reason for rejection was that the CYP was instead recommended for a paediatric/developmental assessment, or an ASD assessment. The third most common reason was that the CYP was signposted to another more appropriate service (including social care/paediatrics). Those who were rejected from CAMHS on the basis of the severity of their presentation were significantly older than those who were rejected for other reasons (t[315] = 3.019, *p* = .003) (12.45 years vs. 11.02 years respectively). Secondly, significantly more males were rejected from CAMHS on the basis of being under severity threshold in comparison to females (Χ^2^[1] = 7.972, *p* = .005) (Figure 2).

### Eligibility for the youth wellbeing in schools team

Of the referrals rejected to the present CAMHS between September 2019 and September 2020, the review of medical records and the CRP database suggested that almost a third (*N* = 95, 29.97%) would meet the criteria for acceptance to the CYWS team. For a small number of referrals (*n* = 6) eligibility was not able to be determined due to a lack of information required to make that decision. A total of 216 referrals were judged as not suitable for inclusion to the CYWS team from the information provided.

Forty-seven of the CYP rejected to CAMHS on the basis of ‘low severity’ would meet criteria for acceptance to the CYWS team, as would 58 additional CYP rejected for other reasons (Χ^2^[1] = 6.117, *p* = .013). These reasons included: having been referred for autism, paediatric or developmental assessment (*n* = 28); referred to educational psychology (*n* = 3); COVID-19 service pressures (*n* = 12; or were signposted to another more suitable service (*n* = 5).

Of all referrals eligible to the CYWS team, 53 (55.8%) of these CYP were referred to CAMHS via their GP, and the second most common referral route was via the CYP’s education provider (*n* = 20, 21.1%). The remainder of potential cases were referred to CAMHS via community health services (*n* = 12, 12.6%), social services (*n* = 8, 8.4%) and self-referral (*n* = 2, 2.1%).

To determine whether a new referral route would discriminate against certain populations of CYP (thereby skewing ease of accessibility unfairly), the ages of those eligible versus those not eligible were compared. There was a significant difference in the ages of those who were eligible for the CYWS team compared to those who are not eligible (t[309] = −2.656, *p* = .008). The average age of eligible CYP was 10.67 years (±3.794), whereas those not eligible were significantly older (11.96 years (±4.195). There was no significant difference in gender proportions for those deemed eligible for inclusion in the CYWS service compared to those not eligible (Χ^2^[1] = 1.413, *p* = .235).

## Discussion

The findings highlight that around one third of the referrals rejected to CAMHS would meet eligibility criteria for acceptance to the CYWS team, potentially allowing more CYP experiencing difficulties with their mental health to get support. While there would be a significant proportion of CYP who would not meet the threshold for either CAMHS or the embedded CYWS, support may be more appropriate from other low-intensity school based support, such as school counsellors, or via more specialist or online services, which CYP and their families would be signposted to by CAMHS. The results showed that the majority of referrals came from GPs and educational providers, supporting the suggestion that teams of CWPs should be accessible to all schools, in the community and in GP surgeries. There were a variety of reasons for referrals being rejected to the present CAMHS team, with the most common reason being that the CYP’s were not found to have a diagnosable mental health condition, or secondly, did not meet the clinical threshold for intervention. There was no significant difference in gender distribution between those eligible for inclusion to the CYWS team versus exclusion, suggesting that a new treatment pathway would not discriminate CYP by gender. Because of the vast range of ethnic backgrounds in this sample, it was not possible to determine whether there were any statistical differences in ethnicity between those who would be accepted to the CYWS team and those who would be rejected.

### Comparisons with existing literature

The range of rejection reasons were comparable to the findings of Crena-Jennings and Hutchinson (2020), who noted that the most common reason for rejection to services was that the CYP’s condition was ‘not suitable’ for CAMHS intervention (e.g. does not have a diagnosable mental health condition), followed by low clinical severity of their condition (Crena-Jennings & Hutchinson, 2020). Another common reason for referrals being rejected included the opinion that referrals did not fit the remit of the service, and were signposted (Crena-Jennings & Hutchinson, 2020), which was the fourth most common reason for referral rejection in CAMHS in this study. The service may benefit from a future comparison of demographics between those rejected to the service and those accepted, to see whether there is an inequity in access.

Previous research has shown that the majority of CAMHS referrals in the UK between 2011 and 2015 came from GP services (46%) (Edbrooke-Childs & Deighton, 2020). This is comparable to the present study, where GP practices were also the primary referrer. If CYP were able to be seen by CWPs and EMHPs in services like the CYWS, many CYP may be able to access support faster. Previous research has shown that seeing CYP with minimal waiting times also has the potential to increase engagement (Westin et al., 2014) and lead to positive outcomes (Fixsen et al., 2010). Thus, it has been suggested in previous research that timely intervention is vital and a priority for CYP experiencing mental health problems (Carr, 2009; Murphy & Fonagy, 2012; Rocks et al., 2020; Solmi et al., 2021). Previous research has found that found black young people were more likely to be referred to CAMHS via other mental health service, social-care/youth justice services and educational routes relative to primary care and white British CYP (Edbrooke-Childs & Deighton, 2020, Chui et al., 2020). Due to missing data on ethnicity, these comparisons were not possible in this sample, but future research would better be able to assess possible differences in how YP present to the service.

Finally, existing literature has also demonstrated that of 60 public CAMHS providers across England, around one third did not even collect data on the reasons for rejecting referrals, or instead held them in a format that was not reportable (Crena-Jennings & Hutchinson, 2020), bringing into question the interpretability of some of the existing data. There were a number of referrals in the current study where a reason for rejection at the time of decision was missing, however, to minimise the effects of missing data, the authors reviewed the electronic records in order to make an informed clinical decision in retrospect as to the possible reason for rejection.

### Strengths and limitations

A key strength of this original project is that this is the first study to our knowledge which has assessed the feasibility of developing a new, single point of access referral route within CAMHS services, to make full use of clinical practitioners who specialise in CYP mental health, thereby being more beneficial to CYP and promoting equity of access to CAMHS. This pilot study assessed the eligibility of all CYP referrals against CYWS criteria to do this and whether a new treatment pathway in a CAMHS service would be valid and equitable. This study used real service data, which contributed to the generalisability of the findings to other CAMHS services within England. Furthermore, this systematic and thorough process of service development could be used as a model for other services developing or restructuring their referral pathways.

There are however a few limitations to be aware of. Between March 2020 and September 2020, referral patterns were interrupted due to the COVID-19 pandemic, and so may not be fully reflective of pre-pandemic referral patterns. Between 9th April and 8th June 2020, no non-urgent referrals were accepted to the team, referrers were provided with signposting to other forms of support and asked to re-refer to the service if case severity escalated. These results should be interpreted with caution, as it is possible that the effects of the COVID-19 pandemic might have had an impact on these referral and rejection figures, as the peak number of rejections to CAMHS rose over the first lockdown period in the UK (March 2020 to June 2020), before dropping to more average monthly levels. Similarly, referral patterns from different types of services may have varied as a result of the pandemic, with fewer people visiting their GPs, members of the public being advised to not attend A&E unless necessary, and schools being closed for a significant period of time. Additionally, as previously discussed, it is suggested that the COVID-19 pandemic may have had an impact on the mental health of CYP, which could conceivably lead to an increased prevalence of mental health conditions in this population, and thereby an increase in the number of referrals to CAMHS nationwide. A recent report into referral levels since the COVID-19 pandemic has shown that CAMHS referrals have recently risen above the levels they were at before the COVID-19 pandemic, however the number of CYP accessing treatment has not (Children’s Commissioner for England, 2020).

In this service, data is inputted into electronic medical records at the clinician’s discretion, which may lead to some inconsistencies and errors. In some cases, multiple reasons for referral rejection were cited, and it was not always clear what the primary reason was. Therefore, the researchers had to make decisions based on clinical judgement from what information was available on the records and the CRP database. There was also a high proportion of missing data for ethnicity, the current study presents data from ethnic categories which do not map directly onto those measured nationally in the census, however these are the categories in use on the present clinical records system. This makes it difficult to compare the ethnic distributions of the referrals compared to data from the census.

It is important to note that some CYP may have been referred more than once during the studied period of time, therefore the number of referrals reported likely do not represent separate individuals. Despite this, this information is likely to sufficiently represent general patterns in referrals, reasons for rejection and whether referred CYP would be eligible for support from the CYWS team.

### Clinical and research implications

Early intervention can reduce the need for further, more intensive support (Garcia et al., 2006) and can be offered through a variety of community settings, such as GP surgeries and schools, and the government aim to increase mental health provision in these areas (Department of Health and Social Care, 2015; Department of Health and Social Care & Department for Education, 2018). However, as identified by many reports and research studies over recent years (Department of Health and Social Care & Department for Education, 2017; Fraser & Blishen, 2007; Garcia et al., 2006; Marshall et al., 2017) mental health support is not available in all schools and colleges. The UK government aim to incentivise all schools and colleges to identify designated senior leads for mental health by 2025, with the hope that this will increase access to quick advice and consultation for CYP. The UK government aims to support NHS teams, schools and colleges to be able to work together and provide mental health support to CYP, including through the development of peer support trials and preventative programmes (Department of Health and Social Care & Department for Education, 2018). Providing support in school settings may also be more acceptable to CYP due to the perceived reduced stigma associated with the setting (Department of Health and Social Care & Department for Education, 2017). Whilst this roll out across schools takes place, and for as long as an inequity in mental health provision across schools exists, CYWS teams should consider adjusting their referral pathways to support CYP without direct access to school-based support. It is plausible that increasing the capacity of the CYWS team could reduce overall waiting times in the service, and thus increase access to support. Community intervention and community support would enable a faster response to the mental health needs of children and young people. And as previously outlined in the introduction, early intervention could also have a considerable economic and social impact (Crena-Jennings & Hutchinson, 2020; Fonagy et al., 2014). Providing early support may also be beneficial to the parents of CYP experiencing these difficulties (Carr, 2009), therefore enabling them to support their child and reduce the chances of their child needing more intensive or crisis support later in life (Mind et al., n.d). The finding that a significant portion of referrals are rejected due to not meeting clinical threshold indicates that there are many children who are identified as needing support but not meeting inclusion criteria to CAMHS. This highlights the need for early intervention services such as CYWS teams more broadly across CAMHS services.

The present study also has implications for research, namely the importance of thorough data reporting, so that future research on CAMHS referrals and outcome data can be utilised to evaluate services. It is particularly pertinent to ensure that key demographic data points, i.e., ethnicity are completed in electronic medical records to ensure that analysis can be conducted to assess the equity of practice. There are also other factors which may impact access to services which are not reflected in referral data, such as stigma, which has been suggested to be a further barrier to support (Hansen et al., 2021; Radez et al., 2021). Thus, future research into this area should consider the possible influence of stigma on routes into CAMHS services and how this may impact the YPs chances of being accepted or rejected for input.

### Conclusions

In summary, this original pilot study showed that a high proportion of CYP who are being rejected by the present CAMHS would be eligible for assessment and possible treatment under an embedded CYWS team staffed by CWPs and EMHPs. It is therefore recommended that in CAMHS services, a pathway should be implemented so that all CYP, regardless of their school, can have access to the CYWS service (and similar embedded teams nationwide). This would necessitate an increase in capacity to the CYWS to meet this increased demand, and therefore an expansion of the CYWS. Based on previous evidence on the effectiveness of early intervention, this is likely to be more cost effective to CAMHS than expanding the current tier 3 service.
